# PEEP-FiO_2_ table versus EIT to titrate PEEP in mechanically ventilated patients with COVID-19-related ARDS

**DOI:** 10.1186/s13054-022-04135-5

**Published:** 2022-09-12

**Authors:** Peter Somhorst, Philip van der Zee, Henrik Endeman, Diederik Gommers

**Affiliations:** Department of Adult Intensive Care, Erasmus Medical Center, Doctor Molewaterplein 40, 3015 GD Rotterdam, The Netherlands

**Keywords:** COVID-19, Acute respiratory distress syndrome, Mechanical ventilation, Positive end-expiratory pressure, Electrical impedance tomography

## Abstract

**Rationale:**

It is unknown how to titrate positive end-expiratory pressure (PEEP) in patients with COVID-19-related acute respiratory distress syndrome (ARDS). Guidelines recommend the one-size-fits-all PEEP-FiO_2_ table. In this retrospective cohort study, an electrical impedance tomography (EIT)-guided PEEP trial was used to titrate PEEP.

**Objectives:**

To compare baseline PEEP according to the high PEEP-FiO_2_ table and personalized PEEP following an EIT-guided PEEP trial.

**Methods:**

We performed an EIT-guided decremental PEEP trial in patients with moderate-to-severe COVID-19-related ARDS upon intensive care unit admission. PEEP was set at the lowest PEEP above the intersection of curves representing relative alveolar overdistention and collapse. Baseline PEEP was compared with PEEP set according to EIT. We identified patients in whom the EIT-guided PEEP trial resulted in a decrease or increase in PEEP of ≥ 2 cmH_2_O.

**Measurements and main results:**

We performed a PEEP trial in 75 patients. In 23 (31%) patients, PEEP was decreased ≥ 2 cmH_2_O, and in 24 (32%) patients, PEEP was increased ≥ 2 cmH_2_O. Patients in whom PEEP was decreased had improved respiratory mechanics and more overdistention in the non-dependent lung region at higher PEEP levels. These patients also had a lower BMI, longer time between onset of symptoms and intubation, and higher incidence of pulmonary embolism. Oxygenation improved in patients in whom PEEP was increased.

**Conclusions:**

An EIT-guided PEEP trial resulted in a relevant change in PEEP in 63% of patients. These results support the hypothesis that PEEP should be personalized in patients with ARDS.

**Supplementary Information:**

The online version contains supplementary material available at 10.1186/s13054-022-04135-5.

## Introduction

It is unknown how to titrate PEEP in patients with COVID-19-related ARDS. Previous randomized controlled trials in patients with ARDS found that a higher PEEP versus a lower PEEP strategy did not reduce mortality rate [1–3]. However, higher PEEP did reduce mortality rate in patients with severe ARDS and tended to increase mortality rate in the subgroup with mild ARDS [4]. Apparently, there are patient subgroups that benefit from higher PEEP and subgroups that do not benefit from higher PEEP.

Initially, COVID-19-related ARDS was thought to be typical ARDS according to the Berlin definition of ARDS [5]. Later, a phenotype consisting of preserved respiratory system compliance and low lung recruitability was described. The application of lower PEEP was advised [6, 7]. Subsequent studies found that respiratory mechanics of patients with COVID-19-related ARDS and typical ARDS, including respiratory system compliance, were similar between groups. Significant heterogeneity was observed in COVID-19-related ARDS similar to non-COVID ARDS [8, 9]. The high PEEP-FiO_2_ table, currently recommended for the treatment of COVID-19-related ARDS, does not take into account patient heterogeneity [10].

From a pulmonary perspective, PEEP titration is finding a compromise between minimal alveolar overdistention and collapse. The recommended PEEP for lung protective ventilation strategies ranges between 5 cmH_2_O and 24 cmH_2_O [11]. A strategy consisting of recruitment maneuvers and titrated PEEP resulted in increased long-term mortality in patients with ARDS [12]. Several studies failed to find a benefit of a one-size-fits-all PEEP strategy, and more research into tailoring of PEEP to the individual patient is recommended [13]. Therefore, we think it is crucial to quantify the amount of alveolar overdistention and collapse at the bedside among parameters reflecting respiratory mechanics. EIT can be used for detecting and quantify regional alveolar overdistention and collapse and allows for personalized PEEP titration [14, 15] In a recent randomized controlled trial in ARDS patients, He et al. [16] showed titration using EIT resulted in a decoupling between PEEP and FiO_2_, but no difference in long-term outcome compared to a PEEP/FiO_2_ table. Hsu et al. [17] compared PEEP titrated using EIT to PEEP set based on pressure–volume loops. They found the EIT leads to lower PEEP and a higher survival rate.

We retrospectively describe a cohort of patients with COVID-19-related ARDS in whom an EIT-guided PEEP trial was used to personalize PEEP. The aim of this study was to compare PEEP set by EIT and baseline PEEP according to the high PEEP-FiO_2_ table [1]

## Methods

### Study design and inclusion criteria

This is a retrospective analysis of a cohort study conducted between March 1 and June 1 2020 in the general intensive care unit (ICU) of the Erasmus MC, Rotterdam, The Netherlands. The first 15 patients in this study have been described previously [18] All patients that met the following criteria were included: 1. age ≥ 16 years; 2. established COVID-19 following a SARS-CoV-2 positive polymerase chain reaction; 3. moderate-to-severe ARDS according to the Berlin definition of ARDS [5]; and 4. intubated and on controlled mechanical ventilation. The Erasmus MC is a tertiary referral hospital, and some patients were intubated and mechanically ventilated elsewhere before transfer to our ICU. A PEEP trial guided by EIT was performed following admission to the ICU according to our local COVID-19 mechanical ventilation protocol. The PEEP trial was performed once in every patient and was not routinely repeated. We did not perform a PEEP trial if patients had a contra-indication for EIT belt placement (e.g., pacemaker, spinal cord injury), inadequate EIT signal (e.g., thoracic bandages, undrained pneumothorax), or hemodynamic instability (MAP < 60 mmHg despite optimization of fluid status and/or use of vasopressors). The Medical Ethical Committee of the Erasmus MC approved this study. According to Dutch legislation, no informed consent was required for the retrospective use of anonymized patient data.

### Study protocol

All patients were ventilated in pressure control mode. Baseline PEEP was set by the attending clinician. The protocol was prescribed using the high PEEP-FiO_2_ table, but the clinician had the freedom to choose the PEEP and FiO_2_ combination within the limits of the table [1]. Patients were fully sedated (Richmond Agitation-Sedation Scale -5) with continuous intravenous infusion of propofol, midazolam and/or opiates. Persisting spontaneous inspiratory efforts were prevented with increased sedation or neuromuscular blockade (rocuronium). Mean arterial blood pressure (MAP) was measured continuously, and noradrenalin was administered to maintain MAP above 65 mmHg prior to the PEEP trial. The fraction of inspired oxygen (FiO_2_) was titrated to obtain a peripheral oxygen saturation (SpO_2_) between 92 and 95%.

The PEEP trial was guided by one of two EIT devices, based on availability: Pulmovista 500, Dräger, Germany or Enlight 1800, Timpel, Brazil. An EIT belt containing surface electrodes was placed in the transversal plane at the 4th–5th intercostal parasternal space according to manufacturer’s instructions. Regional ventilation data were visualized on screen during the entire PEEP trial without repositioning the EIT belt. The PEEP titration tool of the EIT devices was used to guide PEEP titration.

A decremental PEEP trial was performed starting at the baseline PEEP according to the high PEEP-FiO_2_ table (PEEP_base_). We increased the airway pressure until PEEP was 10 cmH_2_O above PEEP_base_ with a minimum of 24 cmH_2_O, corresponding to the maximum PEEP advised by the high PEEP-FiO_2_ table [1]. The imposed driving pressure (i.e., the difference between PEEP and peak pressure) remained unchanged during the trial. In case of hypotension (MAP < 60 mmHg) or desaturation (SpO_2_ < 88%), PEEP was limited to the highest airway pressure without hypotension or desaturation. We aimed to maintain PEEP for at least one minute in order to establish a constant electrical impedance signal. PEEP was reduced in steps of 2 cmH_2_O every 30 s until continuous EIT monitoring showed evident collapse as compared to maximum PEEP. To confirm a further increase in collapse, PEEP was lowered an additional 2 cmH_2_O. Subsequently, we performed a small recruitment maneuver at the highest PEEP used during the PEEP trial for 30 s. The PEEP titration tools of both EIT devices provided a percentage of relative alveolar overdistention and collapse at every PEEP step. PEEP was set (PEEP_set_) at the lowest PEEP step above the intersection of the curves representing relative alveolar overdistention and collapse, as described previously (see Fig. 2C) [18, 19].

Mechanical ventilation, SpO_2_ and hemodynamic parameters were recorded at PEEP_base_ and after 30 min of PEEP_set_. Plateau airway pressure (Pplat) and total PEEP were measured during an inspiratory and expiratory hold procedure, respectively. We used the last arterial blood gas before and the first arterial blood gas after the decremental PEEP trial for calculation of the PaO_2_/FiO_2_ ratio at PEEP_base_ and PEEP_set_, respectively. Patient characteristics were extracted from the patient information system.

The primary goal of this study was to compare PEEP_base_ with PEEP_set_. Secondary goals were to compare respiratory mechanics and oxygenation before and after the PEEP trial. Subsequently, we identified the patients in whom PEEP_set_ according to the EIT-guided PEEP trial was decreased by ≥ 2 cmH_2_O (PEEP_lower_) or was increased by ≥ 2 cmH_2_O (PEEP_higher_) as compared to PEEP_base_. Patients with a change in PEEP_set_ of less than 2 cmH_2_O as compared to PEEP_base_ were assigned to a third group: PEEP_equal_. The change in percentage of relative alveolar overdistention and collapse were reported between PEEP 24 cmH_2_O and PEEP 12 cmH_2_O, because both PEEP levels were reached during the PEEP trial in 93% (*n* = 70) of patients.

### Statistical analysis

Data were presented as mean (standard deviation), median [25th-75th percentile] or count (percentage). Data were tested for normality using the Shapiro–Wilk test. The Student independent *T* test or Mann–Whitney *U* test was used for the comparison between two groups. A one-way ANOVA or the Kruskal–Wallis test was used for the comparison between three groups. The Student dependent *T* test or Wilcoxon signed-rank test was used to compare changes from baseline within patients. A repeated measures ANOVA or Friedman test was used to compare changes over more than two levels. The Chi-square test was used to compare frequencies. Bonferroni correction was applied to correct for multiple testing. Spearman’s rank correlation coefficient (*ρ*) was used for calculation of correlations between variables. A *p* value of ≤ 0.05 was considered to be statistically significant.

## Results

Seventy-five mechanically ventilated patients with COVID-19-related ARDS were included in this retrospective cohort study. Patients had a median age of 64 years [54–71] and a body mass index (BMI) of 30.4 kg/m^2^ (5.8). Median APACHE IV score at ICU admission was 70 (27) and median time since intubation was 3 days [1–8].

**Fig. 1:**
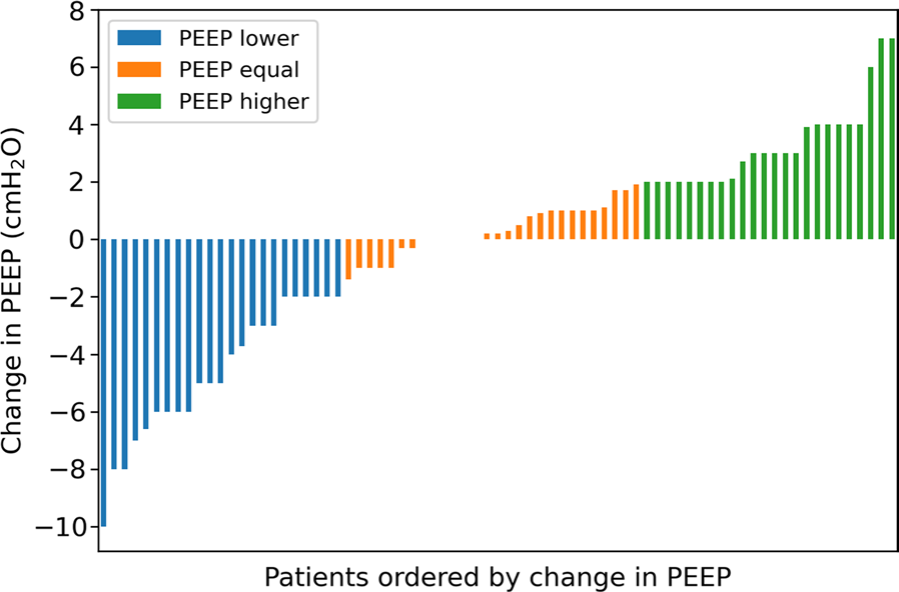
Change in PEEP following the EIT-guided PEEP trial. All 75 patients in this cohort are shown on the *x*-axis. On the *y*-axis, the change in PEEP (PEEP_set_–PEEP_base_) is presented. We identified the patients in which PEEP_set_ according to the EIT-guided PEEP trial was decreased by ≥ 2 cmH_2_O (PEEP_lower_ in blue) or was increased by ≥ 2 cmH_2_O (PEEP_higher_ in green) as compared to PEEP_base_. Patients with a change in PEEP_set_ of less than 2 cmH_2_O as compared to PEEP_base_ were assigned to a third group: PEEP_equal_ (in orange)

**Fig. 2:**
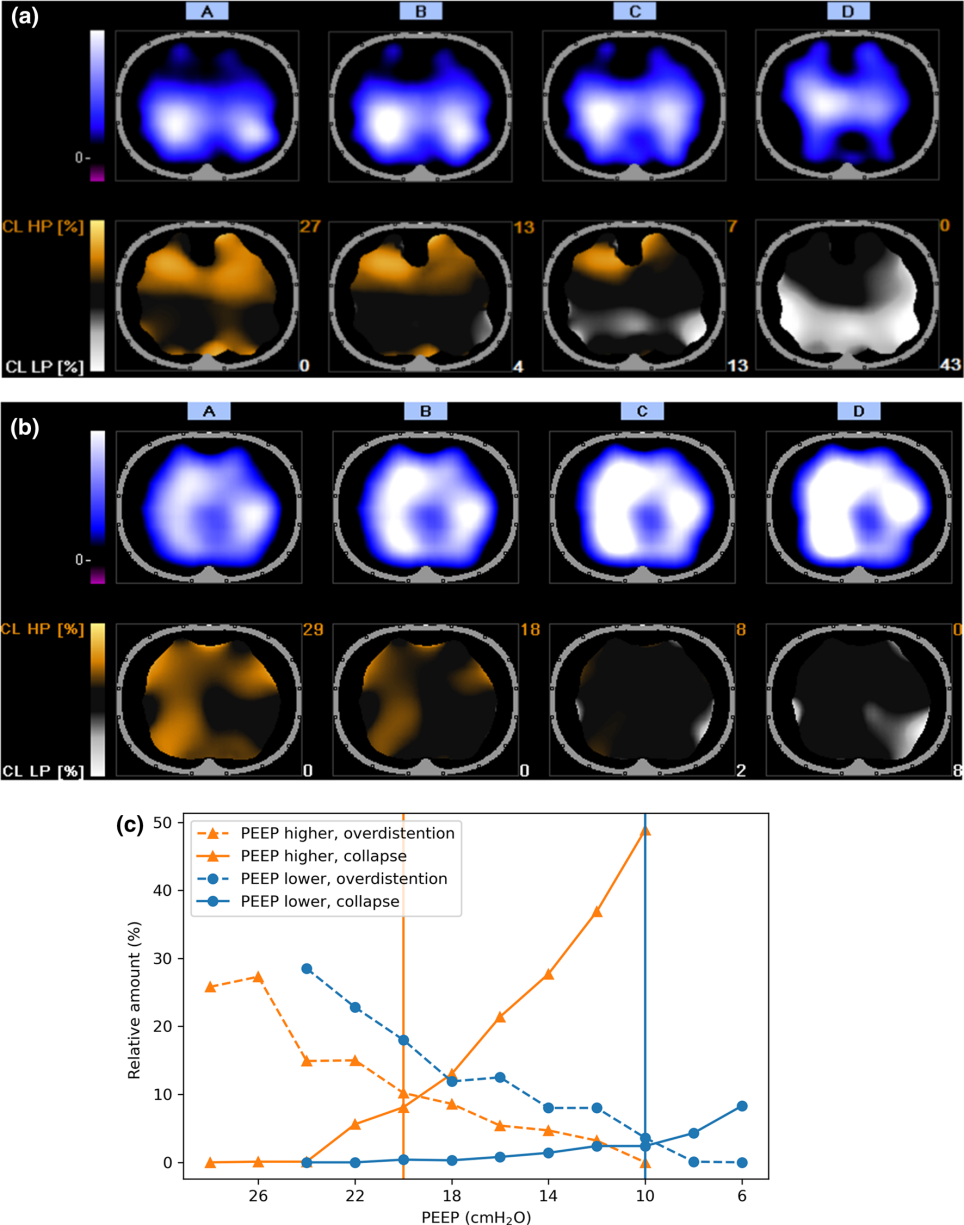
Overdistention and collapse for two typical patients. **a** — A patient assigned to the PEEP_higher_ group. The top row displays ventilation distribution at four levels of PEEP (left to right: 28, 20, 16, and 10 cmH_2_O). Black indicates no ventilation, various shades of blue indicate ventilation, and white indicates the region where most ventilation is detected. At high PEEP levels, ventilation occurs in the dorsal lung regions, whereas the center of ventilation shifts ventrally at lower PEEP levels. The distribution of alveolar overdistention (orange) and alveolar collapse (white) is shown in the bottom row. At high PEEP levels, only a small amount of ventilation is detected in the ventral region as a result of overdistention. At low PEEP levels, ventilation shifts ventrally as a result of alveolar collapse. High PEEP resulted in a relatively small increase in alveolar overdistention and a large decrease in alveolar collapse. This patient was considered to have high recruitability and total PEEP was set at the lowest PEEP step above the intersection of both curves: 20 cmH_2_O., **b** — A patient assigned to the PEEP_lower_ group. The top row displays ventilation distribution at four levels of PEEP (left to right: 24, 20, 12, and 6 cmH_2_O). A decrease in PEEP resulted in an increase in ventilation (light blue to white), and even at a low PEEP level of 6 cmH_2_O, ventilation in the dorsal lung regions is more or less preserved. At high PEEP levels, the relative amount of alveolar overdistention is 29%, which indicates severe alveolar overdistention. In contrast, at low PEEP levels, the amount of alveolar overdistention is significantly reduced, and only a small amount of alveolar collapse is identified (8%). This patient was considered to have low recruitability, and PEEP was set at 10 cmH_2_O., **c** — This plot represents the relative amount of alveolar overdistention and collapse as measured by EIT during a decremental PEEP trial. PEEP was set above at the lowest PEEP step above the intersection of the curves representing relative amount of alveolar overdistention and collapse (vertical lines). The patient in PEEP_higher_ group (orange triangles) had lower amounts of relative overdistention and higher amounts of alveolar collapse at the same PEEP level as compared to the patient assigned to a patient in PEEP_lower_ group (blue dots)

In the entire cohort, we did not observe a difference between the median PEEP level before and after the PEEP trial (Table 1). After the PEEP trial, there was a small increase in static compliance and tidal volume. In 31% of patients (*n* = 23), PEEP was decreased by ≥ 2 cmH_2_O, and in 32% of patients (*n* = 24), PEEP was increased by ≥ 2 cmH_2_O (Fig. 1). The remaining 28 patients (37%) were assigned to PEEP_equal_ group. EIT images of a representative patient from the PEEP_lower_ group and the PEEP_higher_ group are shown in Fig. 2. In five patients (7%), a PEEP of 24 cmH_2_O could not be applied due to desaturation (*n* = 4) or hypotension (*n* = 1). Desaturation occurred only in the PEEP_lower_ group. One (1%) pneumothorax was observed following central catheter placement.

**Table 1:** PEEP_base_ versus PEEP_set_

	PEEP_base_	PEEP_set_	Difference	*p* value
Total PEEP (cmH_2_O)	17.0 [16.0–19.0]	18.0 [14.0–20.0]	0.2 [− 2.0–2.0]	1.00
Plateau pressure (cmH_2_O)	28.0 [25.0–30.8]	28.0 [24.2–30.0]	0.0 [− 3.0–2.0]	0.80
Driving pressure (cmH_2_O)	10.0 [8.0–14.0]	10.0 [7.5–13.0]	− 0.5 [− 1.0–0.8]	0.083
Tidal volume (mL/kg PBW)	6.5 [5.7–7.0]	6.6 [5.9–7.4]	0.2 [− 0.1–0.6]	0.002*
Static compliance (mL/cmH_2_O)	45 [33–59]	49 [35–64]	4 [− 2–8]	0.016*
PaO_2_ (mmHg)	81 [72–93]	80 [68–96]	0 [− 16–13]	1.00
PaO_2_/FiO_2_ ratio (mmHg)	162 [110–201]	159 [123–212]	0 [− 24–51]	0.92
SpO_2_ (%)	95 [93–95]	95 [93–96]	0 [− 2–2]	1.00
PaCO_2_ (mmHg)	45 [41–52]	45 [40–53]	− 1 [− 5–5]	0.71
Systolic blood pressure (mmHg)	130 (24)	134 (23)	4 (27)	0.63
Diastolic blood pressure (mmHg)	60 [54–65]	61 [54–67]	− 1 [− 4–3]	1.00
Mean arterial pressure (mmHg)	82 [76–91]	83 [77–93]	− 1 [− 6–5]	1.00
Heart rate (/min)	79 [70–94]	81 [70–92]	1 [− 2–4]	0.22

Data are presented as mean (standard deviation) or median [25th and 75th percentile]. *p < 0.05

Patients in the PEEP_lower_ group had a lower BMI, a higher APACHE IV score, a longer time between onset of symptoms and intubation, a higher d-dimer concentration at ICU admission, and a higher incidence of pulmonary embolism as compared to the other groups (Table 2). In the entire cohort, we found a statistically significant correlation between PEEP_set_ and BMI (*ρ* = 0.59, *p* value < 0.001), and time between onset of symptoms and intubation (*ρ* = − 0.42, *p* value < 0.001). We did not observe a correlation between PEEP_set_ and APACHE IV score (*ρ* = − 0.10, *p* value 0.41) or *d*-dimer concentration (*ρ* = − 0.06, *p* value 0.66). PEEP_set_ resulted in an increase in tidal volume at the same driving pressure (static compliance was not significantly changed after Bonferroni correction) compared to PEEP_base_ in patients in the PEEP_lower_ group, but not in the other two groups (Table 3). In addition, we observed a significant reduction in plateau pressure in the PEEP_lower_ group. In the patients assigned to PEEP_higher_ group, we observed a significant increase in plateau pressure. There was a trend for a higher PaO_2_/FiO_2_ ratio, but this was not significant after Bonferroni correction.

**Table 2:** Comparison of baseline characteristics between PEEP groups

	Total (*n* = 75)	PEEP_lower_ (*n* = 23)	PEEP_equal_ (*n* = 28)	PEEP_higher_ (*n* = 24)	*p* value
Male gender	59 (79%)	19 (83%)	22 (79%)	18 (75%)	0.82
BMI (kg/m^2^)	30.4 (5.8)	27.8 (5.6)	31.4 (5.5)	31.6 (5.8)	0.037*
Age (y)	64 [54–71]	66 [60–73]	64 [54–68]	59 [53–70]	0.27
Apache IV score at ICU admission	70 (27)	85 (32)	66 (24)	61 (19)	0.004*
Time since onset symptoms (*d*)	14 [10–17]	15 [14–26]	14 [8–17]	12 [7–16]	0.061
Time since intubation (*d*)	3 [1–8]	4 [2–14]	3 [2–6]	2 [1–7]	0.52
Time between onset symptoms and intubation (*d*)	10 [7–12]	10 [9–13]	7 [6–12]	8 [6–10]	0.046*
Time ventilated in other hospital (*d*)	1 [0–4]	2 [0–6]	1 [0–3]	1 [0–3]	0.51
28-day mortality	22 (29%)	8 (35%)	7 (25%)	7 (29%)	0.75
D-dimer at admission (mg/L)	1.6 [0.9–3.5]	2.9 [1.5–8.8]	1.2 [0.7–2.8]	1.3 [1.0–2.2]	0.026*
Pulmonary embolism at PEEP trial	13 (17%)	6 (26%)	3 (11%)	4 (17%)	0.35
Proven pulmonary embolism during ICU admission	38 (51%)	17 (74%)	12 (43%)	9 (38%)	0.026*

Data are presented as mean (standard deviation), count (%) or median [25th and 75th percentile]. *p < 0.05

**Table 3:** Comparison of respiratory mechanics between PEEP groups

		PEEP_lower_ (*n* = 23)	*p* value	PEEP_equal_ (*n* = 28)	*p* value	PEEP_higher_ (*n* = 24)	*p* value
Total PEEP (cmH_2_O)	PEEP_base_	18.0 [16.4–19.0]		17.0 [15.8–19.6]		17.0 [15.8–18.2]	
	PEEP_set_	14.0 [11.0–16.0]	< 0.001*	18.0 [15.8–20.0]	0.48	20.0 [18.0–22.7]	< 0.001*
Plateau pressure (cmH_2_O)	PEEP_base_	29.7 (5.0)		27.9 (3.9)		26.5 (3.3)	
	PEEP_set_	23.9 (4.7)	< 0.001*	27.8 (3.8)	1.00	30.1 (4.6)	< 0.001*
Driving pressure (cmH_2_O)	PEEP_base_	11.0 [8.5–14.1]		9.8 [8.0–12.1]		9.0 [8.2–14.0]	
	PEEP_set_	11.0 [7.0–14.0]	0.065	9.5 [7.8–11.9]	0.37	10.0 [9.0–13.0]	1.00
Tidal volume (mL/kg PBW)	PEEP_base_	6.1 (1.2)		6.8 (1.4)		6.5 (0.8)	
	PEEP_set_	6.6 (1.3)	0.002*	7.1 (1.6)	0.060	6.5 (0.8)	1.00
Static compliance (mL/cmH_2_O)	PEEP_base_	45 (26)		53 (23)		47 (16)	
	PEEP_set_	50 (22)	0.19	55 (19)	1.00	47 (16)	1.00
PaO_2_ (mmHg)	PEEP_base_	87 (29)		96 (54)		82 (16)	
	PEEP_set_	74 (19)	0.31	89 (23)	1.00	87 (16)	0.65
PaO_2_/FiO_2_ ratio (mmHg)	PEEP_base_	141 (59)		188 (102)		162 (66)	
	PEEP_set_	149 (64)	1.00	182 (68)	1.00	185 (72)	0.16
SpO_2_ (%)	PEEP_base_	94 [93–95]		95 [94–95]		94 [93–95]	
	PEEP_set_	94 [92–96]	1.00	96 [93–97]	1.00	94 [93–96]	0.73
PaCO_2_ (mmHg)	PEEP_base_	48 [39–57]		44 [39–53]		45 [42–50]	
	PEEP_set_	44 [39–56]	1.00	44 [40–55]	1.00	46 [42–50]	1.00
Systolic Blood Pressure (mmHg)	PEEP_base_	132 (26)		129 (24)		130 (22)	
	PEEP_set_	143 (19)	0.18	128 (24)	1.00	132 (23)	1.00
Diastolic Blood Pressure (mmHg)	PEEP_base_	60 (11)		61 (9)		61 (11)	
	PEEP_set_	63 (13)	0.57	61 (10)	1.00	61 (11)	1.00
Mean Arterial Pressure (mmHg)	PEEP_base_	81 [74–88]		83 [77–93]		82 [76–86]	
	PEEP_set_	89 [80–96]	0.42	81 [76–88]	0.56	83 [76–90]	1.00
Heart rate (/min)	PEEP_base_	83 [70–94]		87 [74–97]		74 [64–80]	
	PEEP_set_	84 [71–94]	1.00	88 [71–95]	1.00	75 [67–84]	0.043*

Data are presented as mean (standard deviation), count (%) or median [25th and 75th percentile]. *p < 0.05

Additional file 1: Tables S1–4 in de Supplementary Materials show the respiratory mechanics at the baseline PEEP, the highest and lowest PEEP during the decremental PEEP trial and the set PEEP immediately after it was set for the entire cohort (Additional file 1: Table S1) and the PEEP_lower_ (Additional file 1: Table S2), PEEP_equal_ (Additional file 1: Table S3) and PEEP_higher_ (Additional file 1: Table S4) groups.

The relative percentages of alveolar collapse and overdistention at PEEP_set_ did not differ between groups (Table 4). In the PEEP_lower_group, relative alveolar collapse increased by 8.3% (3.6–15.4%) in the dependent lung region and relative alveolar overdistention decreased by 28.3% (22.2–43.2%) in the non-dependent lung region as a result of a PEEP decrease from 24 to 12 cmH_2_O. In contrast, in PEEP_higher_ group, this was 24.2% (20.1–29.2%) and 15.1% (4.4–26.2%), respectively.

**Table 4:** Comparison of alveolar collapse and overdistention between PEEP groups

	Total (*n* = 75)	PEEP_lower_ (*n* = 23)	PEEP_equal_ (*n* = 28)	PEEP_higher_ (*n* = 24)	*p* value
*Alveolar collapse*					
Collapse at PEEPset	6.1 (3.6)	5.8 (3.9)	6.8 (3.9)	5.5 (2.8)	0.56
Collapse at PEEP 12 cmH_2_O	17.8 (10.8)	10.2 (7.9)	17.1 (8.8)	24.5 (11.4)	< 0.001*
Collapse at PEEP 24 cmH_2_O	0.0 [0.0–0.6]	0.0 [0.0–0.2]	0.0 [0.0–0.1]	0.4 [0.0–1.1]	0.12
Collapse diff (PEEP 24- > 12)	17.3 (10.3)	9.8 (7.7)	16.3 (8.0)	24.8 (10.2)	< 0.001*
*Overdistention*					
Overdistention at PEEPset	11.3 [5.4–15.4]	8.8 [4.5–13.1]	10.1 [5.9–15.0]	12.8 [10.2–17.2]	0.20
Overdistention at PEEP 12 cmH_2_O	5.1 [2.1–9.2]	9.6 [4.9–15.5]	3.3 [1.4–6.1]	5.1 [3.6–7.7]	0.062
Overdistention at PEEP 24 cmH_2_O	31.3 [26.3–38.4]	37.5 [31.1–56.0]	30.8 [26.3–38.8]	29.6 [20.4–32.6]	0.025*
Hyperdistention diff (PEEP 24- > 12)	− 25.6 [− 31.6–17.0]	− 28.3 [− 43.2–22.2]	− 26.5 [− 35.0–19.1]	− 22.7 [− 28.0–14.0]	0.13

Data are presented as mean (standard deviation) or median [25th and 75th percentile]. *p < 0.05

## Discussion

Based on the EIT-guided PEEP trial, PEEP was decreased in 31% of patients and increased in 32% of patients. We found a significant positive correlation between PEEP_set_ and BMI. Patients in PEEP_lower_ group had improved respiratory mechanics after the PEEP trial, a lower BMI, longer time between onset of symptoms and intubation, and a higher incidence of pulmonary embolism during ICU admission. In patients in the PEEP_lower_ group, an increase in PEEP resulted in major alveolar overdistention and a small amount of recruitment on EIT. In PEEP_higher_ group, we observed a significant increase in plateau pressure and improved oxygenation after the PEEP trial. In addition, an increase in PEEP resulted in significant alveolar recruitment and small amounts of alveolar overdistention on EIT. Hence, the latter group should be considered as recruitable. The PEEP trial was relatively safe, as 5% of patients had a desaturation and 1% of patients was hypotensive during the PEEP trial.

PEEP_set_ resulted in a trend toward improved respiratory mechanics in the PEEP_lower_ group and improved oxygenation in the PEEP_higher_ group. Both an improved driving pressure and improved oxygenation after a change in PEEP are associated with reduced mortality rate in patients with ARDS [20]. Therefore, we should aim to identify the patients that are likely to respond to PEEP, i.e., recruitability.

Recruitability is the amount of collapsed lung tissue that has the potential for reaeration at higher airway pressures [21]. An increase in PEEP in the patients in PEEP_lower_ group resulted in major alveolar overdistention and a small amount of alveolar recruitment, whereas the patients in PEEP_higher_ group had significant alveolar recruitment and less alveolar overdistention. In patients with COVID-19-related ARDS, alveolar recruitment does not necessarily result in an increase in static compliance [22]. Thus, patients in PEEP_lower_ group were considered to have low recruitability, patients in PEEP_equal_ group had intermediate recruitability and patients in PEEP_higher_ group had high recruitability.

Until now, we tended to focus on the identification of patients that had high recruitability [23]. However, it might also be beneficial to identify the patients that have low recruitability and are prone to alveolar overdistention. Patients with low recruitability had a lower BMI, a higher incidence of pulmonary embolism, and a longer time between onset of symptoms and intubation. Patients with obesity have lower transpulmonary pressures and lower end-expiratory long volumes as a result of higher pressure from the chest wall [24]. BMI has a positive correlation with recruitability and the use of higher PEEP, as higher PEEP increases transpulmonary pressure and reduces alveolar collapse [18]. In addition, patients in PEEP_lower_ group had a higher incidence of pulmonary embolism during ICU admission. These findings suggest that disturbed pulmonary perfusion, resulting in a ventilation-perfusion mismatch, caused hypoxemia in these patients. Nevertheless, all patients had a reduced static compliance, possibly leading to disturbed minute ventilation or increased dead space fraction as well [25]. Patients in PEEP_lower_ group had a longer time between onset of symptoms and intubation. This could indicate that these patients may have had some form of patient self-inflicted lung injury or pulmonary fibrosis [26]. Unfortunately, we had too few CT scans at the day of PEEP titration to test this hypothesis. The PEEP trial did not reach a maximum PEEP of 24 cmH_2_O in four (5.3%) patients because of desaturation. These four patients were assigned to the PEEP_lower_ group and had large amounts of alveolar overdistention. Desaturation at high PEEP could be a clear indication of ventilation-perfusion mismatch, likely due to alveolar overdistention.

An observational cohort performed in the Netherlands found a median PEEP titrated by the clinician of 14.0 cmH_2_O (11.0–15.0) [27]. Two small observational cohorts that used EIT to titrate PEEP found a median PEEP of 12.0 cmH_2_O [28, 29]. In our EIT-guided population, we found a higher median PEEP of 18.0 cmH_2_O (14.0–20.0) as compared to the other studies. Explanations are the relatively high BMI in our cohort and long duration of mechanical ventilation in the cohort of Sella et al. [29]. In addition, there is no consensus on how to interpret EIT data obtained during a PEEP trial [14, 30].

In our study, total PEEP was arbitrarily set at the PEEP level above the intersection of the curves representing relative alveolar overdistention and collapse [18, 19]. We chose this method as it is an intuitive and simple approach that can be performed at the bedside, but arguably assumes that both alveolar overdistention and collapse are equally harmful [31]. Both Perrier et al. [28] and Sella et al. [29] chose to set PEEP at the intersection of both curves itself, whereas Franchineau et al. [32] chose to limit alveolar collapse at 15%, independent of alveolar overdistention. The last approach favors alveolar collapse over alveolar overdistention and likely resulted in a lower set PEEP as compared to the method used in this study. Future research should focus on the best approach to titrate PEEP based on EIT data and its association with clinical outcomes.

Previous randomized controlled trials in patients with ARDS compared PEEP titrated using EIT to conventional methods. In patients with mild-to-severe ARDS, He et al. [16] showed EIT resulted in a similar PEEP compared to the PEEP/FiO_2_ table, but was decoupled from FiO_2_. In patients with moderate-to-severe ARDS, Hsu et al. [17] showed PEEP and mortality rate were lower using EIT compared to pressure–volume loops, but mortality rate was high overall (31% in de EIT group and 56% in the control group, versus 21–27% in the study by He et al. [16] and 29% in the current study). In our study, PEEP was not changed on average for the entire cohort after titration using EIT, but was changed with ≥ 2 cmH_2_O in the majority of patients.

This study has several limitations. First, this retrospective analysis was not prespecified in the study protocol and results should be considered hypothesis-generating. The main purpose of this EIT-guided PEEP trial protocol was to improve clinical practice. As a consequence, mechanical ventilation parameters were only recorded at PEEP_base_ and PEEP_set_, limiting a more accurate retrospective analysis of the PEEP trials and EIT data at every PEEP step. A major limitation of this study is the lack of randomization and of the sequence of interventions. All patients received PEEP set by the clinician using the PEEP-FiO_2_ table first and then the EIT-guided PEEP trial. A part of the improvements in oxygenation and respiratory mechanics may be due to the PEEP trial itself, instead of the titration of PEEP_set_. This is noticeable in the changes in respiratory mechanics for the PEEP_equal_ group. Second, only patients with COVID-19-related ARDS were included in this study. Although respiratory mechanics in non-COVID-19-related ARDS and typical ARDS seem to be similar, it is uncertain whether results can be generalized to the non-COVID-19-related ARDS population [8, 9]. Third, maximum and minimum PEEP reached in all trials varied. The estimation of the amount of collapse and overdistention is based on the maximum compliance for each EIT pixel. It is probable or even likely maximum compliance is not reached for all pixels, e.g., due to residual collapse in the dependent lung at the highest PEEP level. Therefore, approximately 0% alveolar collapse at PEEP 24 cmH_2_O does not necessarily mean that application of higher or prolonged airway pressures cannot result in additional alveolar recruitment. Fourth, we performed a PEEP trial with small steps of 2 cmH_2_O and a short step duration of 30 s. Some other studies report larger steps and longer duration for similar PEEP trials [15, 28, 32]. There is a tradeoff between step size, step duration and the time it takes to complete the protocol. After a change in PEEP, respiratory mechanics can change in multiple ways with different time frames. By rapidly changing PEEP, we did not allow for slow effects like slow derecruitment, morphological changes to the abdomen and diaphragm, changing hemodynamics and changes in pO_2_ and pCO_2_. In addition, as a result of the large numbers of patients with COVID-19, we chose a time-efficient study protocol. Fifth, hemodynamic monitoring was limited to continuous measurement of blood pressure and heart rate. PEEP titration is more than balancing alveolar overdistention and collapse, as PEEP influences cardiac output as well [33]. Although the PEEP trials had limited effects on blood pressure and heart rate, we cannot exclude a decrease in cardiac output. In addition, we did not assess pulmonary perfusion with EIT. Hence, EIT-guided PEEP titration might have resulted in optimal ventilation, but not necessarily in an optimal ventilation-perfusion match. Sixth, ventilation distribution assessed by EIT is measured in only a small cross-sectional slice of the lung. Ventilation distribution changes when the EIT belt is placed more cranially or caudally, further complicating EIT-guided PEEP titration [34]. Seventh, we used devices from two manufacturers to perform the EIT measurements. Although the devices apply the same algorithm by Costa et al. [15] to derive the relative collapse and overdistention, results could vary due to differences in belts, reconstruction models and algorithms. Additional file 1: Tables S5–12 in the supplementary materials show the results presented in Tables 1, 2, 3, 4 split by EIT device. Considering the limited data, it seems possible the Timpel Enlight 1800 gives higher values overdistention at high PEEP compared to the Dräger Pulmovista 500. Due to the small amount of measurements with the Timpel device (*n* = 7), we were not able to properly compare the devices. Overall, considering only the measurements with the Dräger device (*n* = 68) does not change our interpretation or conclusions.

In conclusion, a PEEP trial guided by EIT as compared to PEEP titration based on the PEEP-FiO_2_ table resulted in a clinically relevant change in PEEP in 63% of patients with COVID-19-related ARDS. We found a significant positive correlation between set PEEP and BMI. Patients in whom PEEP was decreased had a lower BMI, a longer time between onset of symptoms and intubation, and a higher incidence of pulmonary embolism. Our results support the hypothesis that PEEP should be personalized in patients with COVID-19-related ARDS in order to reduce the total amount of alveolar overdistention and collapse, i.e., too low or too high PEEP.

## Supplementary Information

**Additional file 1**: **Table S1**. PEEP trials of all patients. **Table S2**. PEEP trials of the patients in the PEEPlower group. **Table S3**. PEEP trials of the patients in the PEEPequal group. **Table S4**. PEEP trials of the patients in the PEEPhigher group. **Table S5**. PEEPbase versus PEEPset for patients where EIT-measurements were performed with the Dräger device. **Table S6**. PEEPbase versus PEEPset for patients where EIT-measurements were performed with the Timpel device. **Table S7**. Comparison of baseline characteristics between PEEP groups for patients where EIT-measurements were performed with the Dräger device. **Table S8**. Comparison of baseline characteristics between PEEP groups for patients where EIT-measurements were performed with the Timpel device. **Table S9**. Comparison of alveolar collapse and overdistention between PEEP groups for patients where EIT-measurements were performed with the Dräger device. **Table S10**. Comparison of alveolar collapse and overdistention between PEEP groups for patients where EIT-measurements were performed with the Timpel device. **Table S11**. Comparison of respiratory mechanics between PEEP groups for patients where EIT-measurements were performed with the Dräger device. **Table S12**. Comparison of respiratory mechanics between PEEP groups for patients where EIT-measurements were performed with the Timpel device.

## Data Availability

The datasets used and analyzed during the current study are available from the corresponding author on reasonable request.

## References

[1] Brower RG (2004). Higher versus lower positive end-expiratory pressures in patients with the acute respiratory distress syndrome. N Engl J Med.

[2] Meade MO (2008). Ventilation strategy using low tidal volumes, recruitment maneuvers, and high positive end-expiratory pressure for acute lung injury and acute respiratory distress syndrome: a randomized controlled trial. JAMA.

[3] Mercat A (2008). Positive end-expiratory pressure setting in adults with acute lung injury and acute respiratory distress syndrome: a randomized controlled trial. JAMA.

[4] Briel M (2010). Higher vs lower positive end-expiratory pressure in patients with acute lung injury and acute respiratory distress syndrome: systematic review and meta-analysis. JAMA.

[5] Ranieri VM (2012). Acute respiratory distress syndrome: the berlin definition. JAMA.

[6] Gattinoni L (2020). COVID-19 pneumonia: different respiratory treatments for different phenotypes?. Intensive Care Med.

[7] Marini JJ, Gattinoni L (2020). Management of COVID-19 respiratory distress. JAMA.

[8] Ziehr DR (2020). Respiratory pathophysiology of mechanically ventilated patients with COVID-19: a cohort study. Am J Respir Crit Care Med.

[9] Haudebourg AF (2020). Respiratory mechanics of COVID-19- versus non-COVID-19-associated acute respiratory distress syndrome. Am J Respir Crit Care Med.

[10] Alhazzani W (2020). Surviving sepsis campaign: guidelines on the management of critically ill adults with coronavirus disease 2019 (COVID-19). Intensive Care Med.

[11] Beitler JR (2020). Lung protection in acute respiratory distress syndrome: what should we target?. Curr Opin Crit Care.

[12] Cavalcanti AB (2017). Effect of lung recruitment and titrated positive end-expiratory pressure (PEEP) vs low PEEP on mortality in patients with acute respiratory distress syndrome: a randomized clinical trial. JAMA.

[13] Sahetya SK (2020). Searching for the optimal positive end-expiratory pressure for lung protective ventilation. Curr Opin Crit Care.

[14] Frerichs I (2017). Chest electrical impedance tomography examination, data analysis, terminology, clinical use and recommendations: consensus statement of the TRanslational EIT developmeNt stuDy group. Thorax.

[15] Costa EL (2009). Bedside estimation of recruitable alveolar collapse and hyperdistension by electrical impedance tomography. Intensive Care Med.

[16] He H (2021). Early individualized positive end-expiratory pressure guided by electrical impedance tomography in acute respiratory distress syndrome: a randomized controlled clinical trial. Crit Care.

[17] Hsu HJ (2021). Positive end-expiratory pressure titration with electrical impedance tomography and pressure-volume curve: a randomized trial in moderate to severe ARDS. Physiol Meas.

[18] van der Zee P, Somhorst P, Endeman H, Gommers D (2020). Electrical impedance tomography for positive end-expiratory pressure titration in COVID-19-related acute respiratory distress syndrome. Am J Respir Crit Care Med.

[19] Pereira SM (2018). Individual positive end-expiratory pressure settings optimize intraoperative mechanical ventilation and reduce postoperative atelectasis. Anesthesiology.

[20] Amato MB (2015). Driving pressure and survival in the acute respiratory distress syndrome. N Engl J Med.

[21] Amato MB, Santiago RR (2016). The recruitability paradox. Am J Respir Crit Care Med.

[22] Fossali T (2022). Effects of prone position on lung recruitment and ventilation-perfusion matching in patients with COVID-19 acute respiratory distress syndrome: a combined CT scan/electrical impedance tomography study. Crit Care Med.

[23] van der Zee P, Gommers D (2019). Recruitment maneuvers and higher PEEP, the so-called open lung concept, in patients with ARDS. Crit Care.

[24] Hibbert K, Rice M, Malhotra A (2012). Obesity and ARDS. Chest.

[25] Saha BK (2021). Correlation of respiratory physiologic parameters in mechanically ventilated coronavirus disease 2019 patients. Crit Care Explor.

[26] Brochard L, Slutsky A, Pesenti A (2017). Mechanical ventilation to minimize progression of lung injury in acute respiratory failure. Am J Respir Crit Care Med.

[27] Botta M (2021). Ventilation management and clinical outcomes in invasively ventilated patients with COVID-19 (PRoVENT-COVID): a national, multicentre, observational cohort study. Lancet Respir Med.

[28] Perier F (2020). Electrical impedance tomography to titrate positive end-expiratory pressure in COVID-19 acute respiratory distress syndrome. Crit Care.

[29] Sella N (2020). Positive end-expiratory pressure titration in COVID-19 acute respiratory failure: electrical impedance tomography vs. PEEP/FiO_2_ tables. Crit Care.

[30] van der Zee P, Somhorst P, Endeman H, Gommers D (2020). Reply to van den berg and van der hoeven: in patients with ARDS, optimal PEEP should not be determined using the intersection of relative collapse and relative overdistention. Am J Respir Crit Care Med.

[31] Gattinoni L, Quintel M, Marini JJ (2018). Volutrauma and atelectrauma: which is worse?. Crit Care.

[32] Franchineau G (2017). Bedside contribution of electrical impedance tomography to setting positive end-expiratory pressure for extracorporeal membrane oxygenation-treated patients with severe acute respiratory distress syndrome. Am J Respir Crit Care Med.

[33] Fougeres E (2010). Hemodynamic impact of a positive end-expiratory pressure setting in acute respiratory distress syndrome: importance of the volume status. Crit Care Med.

[34] Bikker IG, Preis C, Egal M, Bakker J, Gommers D (2011). Electrical impedance tomography measured at two thoracic levels can visualize the ventilation distribution changes at the bedside during a decremental positive end-expiratory lung pressure trial. Crit Care.

