# Supplementary material for: PEEP-FiO2 table versus EIT to titrate PEEP in mechanically ventilated patients with COVID-19-related ARDS
Source: Crit Care. 2022 Sep 12;26:272. doi: 10.1186/s13054-022-04135-5 (PMC9465137; doi:10.1186/s13054-022-04135-5)
Supplement: Supplementary file 1 — Additional file 1: Table S1. PEEP trials of all patients. Table S2. PEEP trials of the patients in the PEEPlower group. Table S3. PEEP trials of the patients in the PEEPequal group. Table S4. PEEP trials of the patients in the PEEPhigher group. Table S5. PEEPbase versus PEEPset for patients where EIT-measurements were performed with the Dräger device. Table S6. PEEPbase versus PEEPset for patients where EIT-measurements were performed with the Timpel device. Table S7. Comparison of baseline characteristics between PEEP groups for patients where EIT-measurements were performed with the Dräger device. Table S8. Comparison of baseline characteristics between PEEP groups for patients where EIT-measurements were performed with the Timpel device. Table S9. Comparison of alveolar collapse and overdistention between PEEP groups for patients where EIT-measurements were performed with the Dräger device. Table S10. Comparison of alveolar collapse and overdistention between PEEP groups for patients where EIT-measurements were performed with the Timpel device. Table S11. Comparison of respiratory mechanics between PEEP groups for patients where EIT-measurements were performed with the Dräger device. Table S12. Comparison of respiratory mechanics between PEEP groups for patients where EIT-measurements were performed with the Timpel device. [file 13054_2022_4135_MOESM1_ESM.docx]

# Supplementary materials

Table S1: PEEP trials of all patients.

| All patients | Baseline | Highest PEEP | Lowest PEEP | Set PEEP | p value |
| --- | --- | --- | --- | --- | --- |
| Total PEEP (cmH2O) | 17.0 [16.0-19.0] | 27.0 [26.0-28.0] | 10.2 [7.0-12.0] | 18.0 [14.0-20.0] | <0.001* |
| Plateau pressure (cmH2O) | 28.0 [25.0-30.2] | 38.0 [36.0-42.0] | 19.5 [17.0-22.0] | 28.0 [24.8-30.0] | <0.001* |
| Driving pressure (cmH2O) | 10.0 [8.0-14.0] | 11.5 [9.0-15.2] | 9.0 [6.7-12.2] | 10.0 [7.3-13.0] | <0.001* |
| Tidal volume (mL/kg PBW) | 6.5 [5.7-7.0] | 5.2 [4.5-5.8] | 6.8 [6.0-7.6] | 6.6 [5.9-7.3] | <0.001* |
| Dynamic compliance (mL/cmH2O) | 38 [26-48] | 27 [16-35] | 42 [32-55] | 39 [28-50] | <0.001* |
| FiO2 | 60 [60-70] | 70 [60-85] | 60 [60-70] | 60 [60-70] | 0.26 |
| SpO2 (%) | 95 [93-95] | 95 [92-96] | 94 [92-96] | 95 [93-96] | 0.37 |
| PetCO2 (mmHg) | 35 [30-42] | 38 [32-45] | 35 [31-42] | 35 [31-42] | <0.001* |
| Systolic Blood Pressure (mmHg) | 128 [115-145] | 115 [99-136] | 136 [124-150] | 131 [116-150] | <0.001* |
| Diastolic Blood Pressure (mmHg) | 60 [54-66] | 55 [49-61] | 61 [55-69] | 61 [54-66] | <0.001* |
| Mean Arterial Pressure (mmHg) | 83 [77-91] | 75 [69-83] | 85 [80-96] | 83 [76-92] | <0.001* |
| Heart rate (/min) | 78 [70-93] | 82 [68-93] | 80 [69-91] | 80 [69-92] | 0.52 |

Data are presented as median and 25th and 75th percentile.

Table S2: PEEP trials of the patients in the PEEP_lower_ group.

| PEEP_lower_ (n=23) | Baseline | Highest PEEP | Lowest PEEP | Set PEEP | p value |
| --- | --- | --- | --- | --- | --- |
| Total PEEP (cmH2O) | 18.0 [16.2-19.0] | 26.0 [26.0-28.0] | 9.6 [6.8-11.0] | 14.0 [11.2-16.0] | <0.001* |
| Plateau pressure (cmH2O) | 28.5 [26.2-32.8] | 39.0 [36.0-41.8] | 19.0 [16.0-21.0] | 24.5 [21.2-28.0] | <0.001* |
| Driving pressure (cmH2O) | 11.0 [8.2-14.0] | 12.5 [9.0-16.0] | 10.0 [7.2-12.8] | 11.5 [7.0-14.0] | <0.001* |
| Tidal volume (mL/kg PBW) | 6.2 [5.2-6.8] | 5.1 [3.8-5.7] | 6.9 [5.7-7.4] | 6.6 [5.5-7.4] | <0.001* |
| Dynamic compliance (mL/cmH2O) | 32 [22-47] | 21 [14-35] | 38 [28-51] | 32 [24-45] | <0.001* |
| FiO2 | 40 [40-40] | 40 [40-40] | 40 [40-40] | 40 [40-40] | 1.00 |
| SpO2 (%) | 94 [93-95] | 93 [88-95] | 95 [93-96] | 94 [92-96] | 0.029* |
| PetCO2 (mmHg) | 35 [29-44] | 34 [31-44] | 32 [29-43] | 34 [29-39] | 0.032* |
| Systolic Blood Pressure (mmHg) | 136 [121-145] | 115 [107-140] | 145 [135-164] | 143 [131-157] | <0.001* |
| Diastolic Blood Pressure (mmHg) | 61 [50-66] | 51 [48-61] | 62 [56-70] | 62 [54-70] | 0.012* |
| Mean Arterial Pressure (mmHg) | 85 [80-89] | 72 [67-87] | 92 [85-99] | 89 [80-95] | <0.001* |
| Heart rate (/min) | 83 [69-88] | 84 [70-88] | 84 [69-88] | 81 [71-91] | 0.90 |

Data are presented as median and 25th and 75th percentile.

Table S3: PEEP trials of the patients in the PEEP_equal_ group.

| PEEP_equal_ (n=28) | Baseline | Highest PEEP | Lowest PEEP | Set PEEP | p value |
| --- | --- | --- | --- | --- | --- |
| Total PEEP (cmH2O) | 17.0 [15.5-19.8] | 26.7 [25.5-28.0] | 11.0 [8.0-13.9] | 18.0 [15.5-20.0] | <0.001* |
| Plateau pressure (cmH2O) | 28.0 [25.5-30.5] | 38.0 [36.0-41.0] | 20.0 [18.0-22.0] | 28.0 [26.0-30.0] | <0.001* |
| Driving pressure (cmH2O) | 10.0 [8.1-12.2] | 11.0 [9.2-13.5] | 9.0 [6.3-11.0] | 9.7 [7.5-11.9] | <0.001* |
| Tidal volume (mL/kg PBW) | 6.5 [5.7-7.3] | 5.2 [4.9-5.7] | 6.6 [6.0-7.6] | 7.0 [6.0-7.5] | <0.001* |
| Dynamic compliance (mL/cmH2O) | 43 [33-51] | 28 [25-38] | 46 [39-57] | 46 [37-54] | <0.001* |
| FiO2 | 60 [60-60] | 72 [66-79] | 60 [60-60] | 60 [60-60] | 0.39 |
| SpO2 (%) | 95 [94-95] | 95 [94-96] | 94 [91-96] | 95 [93-96] | 0.081 |
| PetCO2 (mmHg) | 35 [31-46] | 39 [33-47] | 35 [32-44] | 35 [33-43] | 0.031* |
| Systolic Blood Pressure (mmHg) | 126 [110-142] | 114 [99-132] | 134 [120-141] | 128 [112-144] | <0.001* |
| Diastolic Blood Pressure (mmHg) | 61 [54-65] | 54 [51-60] | 59 [54-65] | 59 [55-63] | 0.003* |
| Mean Arterial Pressure (mmHg) | 83 [77-93] | 75 [68-84] | 82 [79-90] | 81 [76-88] | <0.001* |
| Heart rate (/min) | 88 [74-99] | 89 [72-101] | 86 [72-97] | 89 [72-96] | 0.80 |

Data are presented as median and 25th and 75th percentile.

Table S4: PEEP trials of the patients in the PEEP_higher_ group.

| PEEP_higher_ (n=24) | Baseline | Highest PEEP | Lowest PEEP | Set PEEP | p value |
| --- | --- | --- | --- | --- | --- |
| Total PEEP (cmH2O) | 17.0 [15.8-18.2] | 27.0 [25.8-29.0] | 10.0 [7.0-12.5] | 20.0 [18.0-22.7] | <0.001* |
| Plateau pressure (cmH2O) | 26.0 [24.5-28.5] | 38.0 [36.0-43.0] | 18.0 [17.0-22.5] | 30.0 [28.0-32.5] | <0.001* |
| Driving pressure (cmH2O) | 9.0 [8.2-14.0] | 11.0 [10.0-16.0] | 8.7 [7.0-13.0] | 10.0 [9.0-13.0] | <0.001* |
| Tidal volume (mL/kg PBW) | 6.5 [6.0-6.9] | 5.1 [4.4-6.3] | 6.6 [6.1-7.3] | 6.5 [6.0-7.1] | <0.001* |
| Dynamic compliance (mL/cmH2O) | 38 [26-44] | 27 [16-34] | 41 [33-48] | 39 [28-45] | <0.001* |
| FiO2 | 80 [75-85] | 85 [78-92] | 85 [78-92] | 85 [78-92] | 0.39 |
| SpO2 (%) | 94 [93-95] | 95 [92-96] | 94 [92-97] | 94 [93-96] | 0.84 |
| PetCO2 (mmHg) | 35 [30-38] | 39 [35-43] | 36 [32-39] | 37 [33-41] | 0.004* |
| Systolic Blood Pressure (mmHg) | 125 [115-145] | 109 [99-130] | 134 [124-148] | 128 [114-147] | <0.001* |
| Diastolic Blood Pressure (mmHg) | 59 [55-66] | 55 [52-62] | 61 [54-69] | 62 [54-66] | 0.007* |
| Mean Arterial Pressure (mmHg) | 82 [76-86] | 75 [70-82] | 85 [80-96] | 83 [76-90] | 0.002* |
| Heart rate (/min) | 74 [64-80] | 74 [63-84] | 75 [64-86] | 75 [67-84] | 0.12 |

Data are presented as median and 25th and 75th percentile.

Table S5: PEEP_base_ versus PEEP_set_ for patients where EIT-measurements were performed with the Dräger device.

|  | PEEP_base_ | PEEP_set_ | Difference | p-value |
| --- | --- | --- | --- | --- |
| Total PEEP (cmH2O) | 17.0 [16.0-19.0] | 18.0 [14.0-20.9] | 0.7 [-2.0-2.0] | 1.00 |
| Plateau pressure (cmH2O) | 28.0 (4.1) | 27.4 (5.1) | -0.5 (4.6) | 1.00 |
| Driving pressure (cmH2O) | 10.0 [8.1-14.0] | 10.0 [7.7-13.0] | -1.0 [-1.0-0.7] | 0.080 |
| Tidal volume (mL/kg PBW) | 6.5 [5.7-7.0] | 6.6 [6.0-7.3] | 0.2 [-0.1-0.7] | 0.002* |
| Static compliance (mL/cmH2O) | 44 [33-60] | 49 [34-64] | 4 [-2-8] | 0.035* |
| PaO2 (mmHg) | 80 [73-92] | 80 [68-95] | 0 [-16-13] | 1.00 |
| PaO2/FiO2 ratio (mmHg) | 161 [113-199] | 161 [123-211] | 0 [-25-55] | 0.90 |
| SpO2 (%) | 95 [93-95] | 94 [93-96] | 0 [-2-2] | 1.00 |
| PaCO2 (mmHg) | 45 [40-53] | 45 [40-52] | -1 [-5-4] | 0.57 |
| Systolic Blood Pressure (mmHg) | 126 [115-145] | 131 [114-147] | -2 [-10-12] | 1.00 |
| Diastolic Blood Pressure (mmHg) | 60 [54-65] | 60 [54-66] | -1 [-4-3] | 1.00 |
| Mean Arterial Pressure (mmHg) | 83 [76-91] | 83 [76-91] | -1 [-6-5] | 1.00 |
| Heart rate (/min) | 83 [72-95] | 81 [70-92] | 1 [-1-4] | 0.11 |

Data are presented as mean (standard deviation) or median [25th and 75th percentile]. * p < 0.05

Table S6: PEEP_base_ versus PEEP_set_ for patients where EIT-measurements were performed with the Timpel device.

|  | PEEP_base_ | PEEP_set_ | Difference | p-value |
| --- | --- | --- | --- | --- |
| Total PEEP (cmH2O) | 18.0 [15.8-19.8] | 17.0 [14.0-18.6] | -2.0 [-3.5-0.0] | 0.47 |
| Plateau pressure (cmH2O) | 28.0 [23.5-30.5] | 26.0 [25.0-27.5] | -3.0 [-3.5-0.5] | 0.47 |
| Driving pressure (cmH2O) | 11.0 [7.2-11.8] | 9.0 [7.5-11.9] | 0.0 [-0.8-0.5] | 1 |
| Tidal volume (mL/kg PBW) | 6.2 [5.7-7.3] | 6.2 [5.5-7.4] | -0.0 [-0.0-0.1] | 1 |
| Static compliance (mL/cmH2O) | 51 [37-55] | 52 [38-66] | 4 [0-5] | 0.66 |
| PaO2 (mmHg) | 83 [72-93] | 71 [66-107] | 6 [-13-24] | 1 |
| PaO2/FiO2 ratio (mmHg) | 164 [114-216] | 159 [112-222] | 7 [-18-11] | 1 |
| SpO2 (%) | 96 [94-97] | 96 [94-96] | -1 [-1-2] | 1 |
| PaCO2 (mmHg) | 44 [42-48] | 45 [39-53] | 2 [-5-7] | 0.69 |
| Systolic Blood Pressure (mmHg) | 129 [103-156] | 152 [146-164] | 4 [-5-54] | 1 |
| Diastolic Blood Pressure (mmHg) | 52 [49-71] | 65 [50-70] | 3 [-2-14] | 1 |
| Mean Arterial Pressure (mmHg) | 81 [75-84] | 92 [84-100] | 3 [-3-29] | 1 |
| Heart rate (/min) | 75 [65-82] | 71 [62-90] | -3 [-4-0] | 0.46 |

Data are presented as mean (standard deviation) or median [25th and 75th percentile]. * p < 0.05

Table S7: Comparison of baseline characteristics between PEEP groups for patients where EIT-measurements were performed with the Dräger device.

|  | Total (n=68) | PEEP_lower_ (n=19) | PEEP_equal_ (n=25) | PEEP_higher_ (n=24) | p-value |
| --- | --- | --- | --- | --- | --- |
| Male gender | 53 (78%) | 15 (79%) | 20 (80%) | 18 (75%) | 0.91 |
| BMI (kg/m2) | 30.5 (6.0) | 27.3 (5.7) | 31.8 (5.6) | 31.6 (5.8) | 0.020* |
| Age (y) | 64 [56-70] | 66 [62-73] | 63 [54-68] | 59 [53-70] | 0.15 |
| Apache IV score at ICU admission | 69 (27) | 84 (32) | 66 (26) | 61 (19) | 0.013* |
| Time since onset symptoms (d) | 14 [10-18] | 15 [14-27] | 15 [8-18] | 12 [7-16] | 0.037* |
| Time since intubation (d) | 3 [2-9] | 4 [2-16] | 3 [2-7] | 2 [1-7] | 0.48 |
| Time between onset symptoms and intubation (d) | 10 [7-12] | 11 [10-13] | 7 [6-12] | 8 [6-10] | 0.056 |
| Time ventilated in other hospital (d) | 1 [0-3] | 2 [0-8] | 1 [0-3] | 1 [0-3] | 0.71 |
| 28 day mortality | 21 (31%) | 7 (37%) | 7 (28%) | 7 (29%) | 0.80 |
| D-dimer at admission (mg/L) | 1.5 [0.9-3.2] | 2.9 [1.3-9.3] | 1.2 [0.7-2.8] | 1.3 [1.0-2.2] | 0.090 |
| Pulmonary embolism at PEEP trial | 12 (18%) | 5 (26%) | 3 (12%) | 4 (17%) | 0.46 |
| Proven pulmonary embolism during ICU admission | 32 (47%) | 13 (68%) | 10 (40%) | 9 (38%) | 0.088 |

Data are presented as mean (standard deviation), count (%) or median [25th and 75th percentile]. * p < 0.05

Table S8: Comparison of baseline characteristics between PEEP groups for patients where EIT-measurements were performed with the Timpel device.

|  | Total (n=7) | PEEP_lower_ (n=4) | PEEP_equal_ (n=3) | p-value |
| --- | --- | --- | --- | --- |
| Male gender | 6 (86%) | 4 (100%) | 2 (67%) | 0.88 |
| BMI (kg/m2) | 27.8 [26.9-29.7] | 28.1 [27.7-30.8] | 26.3 [26.2-28.6] | 0.40 |
| Age (y) | 65 [53-72] | 58 [49-68] | 71 [62-72] | 0.63 |
| Apache IV score at ICU admission | 74 [58-90] | 88 [70-110] | 59 [58-68] | 0.48 |
| Time since onset symptoms (d) | 9 [5-12] | 13 [12-14] | 4 [4-6] | 0.077 |
| Time since intubation (d) | 2 [1-4] | 4 [2-4] | 1 [1-2] | 0.20 |
| Time between onset symptoms and intubation (d) | 7 [4-8] | 9 [8-10] | 3 [2-5] | 0.12 |
| Time ventilated in other hospital (d) | 1 [0-3] | 3 [2-4] | 1 [0-1] | 0.28 |
| 28 day mortality | 1 (14%) | 1 (25%) | 0 (0%) | 1.00 |
| D-dimer at admission (mg/L) | 4.2 [3.3-6.4] | 4.2 [3.3-6.4] | n.d.a. |  |
| Pulmonary embolism at PEEP trial | 1 (14%) | 1 (25%) | 0 (0%) | 1.00 |
| Proven pulmonary embolism during ICU admission | 6 (86%) | 4 (100%) | 2 (67%) | 0.88 |

Data are presented as mean (standard deviation), count (%) or median [25th and 75th percentile]. * p < 0.05, n.d.a.: no data available

Table S9: Comparison of alveolar collapse and overdistention between PEEP groups for patients where EIT-measurements were performed with the Dräger device.

|  | Total (n=68) | PEEP_lower_ (n=19) | PEEP_equal_ (n=25) | PEEP_higher_ (n=24) | p-value |
| --- | --- | --- | --- | --- | --- |
| **Alveolar collapse** |  |  |  |  |  |
| Collapse at PEEPset | 6.4 (3.2) | 5.8 (2.9) | 7.6 (3.5) | 5.5 (2.8) | 0.17 |
| Collapse at PEEP 12 cmH2O | 18.5 (10.9) | 10.7 (7.1) | 16.7 (9.2) | 24.5 (11.4) | 0.004* |
| Collapse at PEEP 24 cmH2O | 0.0 [0.0-0.8] | 0.0 [0.0-0.2] | 0.0 [0.0-0.2] | 0.4 [0.0-1.1] | 0.19 |
| Collapse diff (PEEP 24->12) | 17.9 (10.4) | 10.1 (7.2) | 15.8 (8.3) | 24.8 (10.2) | <0.001* |
| **Overdistention** |  |  |  |  |  |
| Overdistention at PEEPset | 11.1 (6.9) | 8.8 (6.6) | 10.9 (6.5) | 13.8 (7.3) | 0.20 |
| Overdistention at PEEP 12 cmH2O | 5.1 [2.4-9.2] | 9.6 [5.3-15.5] | 3.6 [1.6-6.2] | 5.1 [3.6-7.7] | 0.055 |
| Overdistention at PEEP 24 cmH2O | 31.1 [25.8-33.9] | 31.8 [31.1-43.8] | 30.7 [26.3-38.4] | 29.6 [20.4-32.6] | 0.20 |
| Hyperdistention diff (PEEP 24->12) | -23.2 [-28.7–16.2] | -23.2 [-28.3–11.4] | -25.7 [-31.2–19.1] | -22.7 [-28.0–14.0] | 0.35 |

Data are presented as mean (standard deviation) or median [25th and 75th percentile]. * p < 0.05

Table S10: Comparison of alveolar collapse and overdistention between PEEP groups for patients where EIT-measurements were performed with the Timpel device.

|  | Total (n=7) | PEEP_lower_ (n=4) | PEEP_equal_ (n=3) | p-value |
| --- | --- | --- | --- | --- |
| **Alveolar collapse** |  |  |  |  |
| Collapse at PEEPset | 1.2 [0.8-6.8] | 3.6 [1.0-8.3] | 1.2 [0.7-4.4] | 0.72 |
| Collapse at PEEP 12 cmH2O | 12.6 [5.6-22.6] | 5.6 [2.1-12.6] | 21.1 [16.9-23.3] | 0.23 |
| Collapse at PEEP 24 cmH2O | 0.0 [0.0-0.1] | 0.0 [0.0-0.2] | 0.0 [0.0-0.1] | 1 |
| Collapse diff (PEEP 24->12) | 12.6 [5.6-22.1] | 5.6 [2.1-12.4] | 21.0 [16.8-23.2] | 0.23 |
| **Overdistention** |  |  |  |  |
| Overdistention at PEEPset | 9.7 [6.8-14.2] | 9.6 [7.4-11.0] | 13.4 [8.8-19.9] | 0.63 |
| Overdistention at PEEP 12 cmH2O | 2.5 [0.6-7.7] | 6.1 [1.9-15.1] | 1.1 [0.6-3.4] | 0.48 |
| Overdistention at PEEP 24 cmH2O | 45.0 [40.4-57.6] | 49.6 [41.8-57.7] | 45.0 [33.0-52.1] | 0.86 |
| Hyperdistention diff (PEEP 24->12) | -39.3 [-44.8–33.1] | -39.1 [-44.0–34.0] | -39.3 [-48.8–30.1] | 1 |

Data are presented as mean (standard deviation) or median [25th and 75th percentile]. * p < 0.05

Table S11: Comparison of respiratory mechanics between PEEP groups for patients where EIT-measurements were performed with the Dräger device.

|  |  | PEEP_lower_ (n=19) | p-value | PEEP_equal_ (n=25) | p-value | PEEP_higher_ (n=24) | p-value |
| --- | --- | --- | --- | --- | --- | --- | --- |
| Total PEEP (cmH2O) | PEEP_base_ | 18.0 [16.4-19.0] |  | 17.0 [16.0-20.0] |  | 17.0 [15.8-18.2] |  |
|  | PEEP_set_ | 14.0 [9.7-15.5] | <0.001* | 18.0 [16.0-20.0] | 0.67 | 20.0 [18.0-22.7] | <0.001* |
| Plateau pressure (cmH2O) | PEEP_base_ | 29.6 (4.8) |  | 28.0 (3.8) |  | 26.5 (3.3) |  |
|  | PEEP_set_ | 23.7 (4.8) | <0.001* | 27.8 (3.9) | 1.00 | 30.1 (4.6) | <0.001* |
| Driving pressure (cmH2O) | PEEP_base_ | 13.0 [9.0-15.1] |  | 9.5 [8.0-12.0] |  | 9.0 [8.2-14.0] |  |
|  | PEEP_set_ | 12.0 [7.0-14.5] | 0.13 | 9.3 [7.0-11.8] | 0.14 | 10.0 [9.0-13.0] | 1.00 |
| Tidal volume (mL/kg PBW) | PEEP_base_ | 6.1 (1.1) |  | 6.8 (1.5) |  | 6.5 (0.8) |  |
|  | PEEP_set_ | 6.7 (1.2) | 0.001* | 7.1 (1.7) | 0.067 | 6.5 (0.8) | 1.00 |
| Static compliance (mL/cmH2O) | PEEP_base_ | 43 (27) |  | 54 (23) |  | 47 (16) |  |
|  | PEEP_set_ | 48 (22) | 0.42 | 57 (19) | 1.00 | 47 (16) | 1.00 |
| PaO2 (mmHg) | PEEP_base_ | 91 (30) |  | 96 (57) |  | 82 (16) |  |
|  | PEEP_set_ | 75 (20) | 0.36 | 86 (21) | 1.00 | 87 (16) | 0.65 |
| PaO2/FiO2 ratio (mmHg) | PEEP_base_ | 145 (56) |  | 186 (107) |  | 162 (66) |  |
|  | PEEP_set_ | 157 (65) | 1.00 | 175 (67) | 1.00 | 185 (72) | 0.16 |
| SpO2 (%) | PEEP_base_ | 94 [93-95] |  | 95 [94-95] |  | 94 [93-95] |  |
|  | PEEP_set_ | 93 [92-96] | 1.00 | 95 [93-97] | 1.00 | 94 [93-96] | 0.73 |
| PaCO2 (mmHg) | PEEP_base_ | 47 [36-57] |  | 45 [39-53] |  | 45 [42-50] |  |
|  | PEEP_set_ | 44 [38-53] | 1.00 | 45 [41-56] | 1.00 | 46 [42-50] | 1.00 |
| Systolic Blood Pressure (mmHg) | PEEP_base_ | 131 (23) |  | 130 (24) |  | 130 (22) |  |
|  | PEEP_set_ | 142 (19) | 0.077 | 126 (24) | 1.00 | 132 (23) | 1.00 |
| Diastolic Blood Pressure (mmHg) | PEEP_base_ | 59 (9) |  | 62 (8) |  | 61 (11) |  |
|  | PEEP_set_ | 63 (11) | 0.28 | 60 (10) | 1.00 | 61 (11) | 1.00 |
| Mean Arterial Pressure (mmHg) | PEEP_base_ | 81 [74-88] |  | 83 [77-94] |  | 82 [76-86] |  |
|  | PEEP_set_ | 89 [80-96] | 0.16 | 80 [76-84] | 0.12 | 83 [76-90] | 1.00 |
| Heart rate (/min) | PEEP_base_ | 83 [72-95] |  | 88 [74-95] |  | 74 [64-80] |  |
|  | PEEP_set_ | 84 [74-94] | 1.00 | 89 [73-94] | 1.00 | 75 [67-84] | 0.043* |

Data are presented as mean (standard deviation), count (%) or median [25th and 75th percentile]. * p < 0.05

Table S12: Comparison of respiratory mechanics between PEEP groups for patients where EIT-measurements were performed with the Timpel device.

|  |  | PEEP_lower_ (n=4) | p-value | PEEP_equal_ (n=3) | p-value |
| --- | --- | --- | --- | --- | --- |
| Total PEEP (cmH2O) | PEEP_base_ | 19.0 [17.2-22.0] |  | 16.7 [14.8-18.1] |  |
|  | PEEP_set_ | 16.0 [13.5-18.5] | 0.38 | 17.0 [15.5-18.1] | 1.00 |
| Plateau pressure (cmH2O) | PEEP_base_ | 28.5 [26.8-31.5] |  | 24.0 [23.5-28.0] |  |
|  | PEEP_set_ | 25.5 [23.5-26.8] | 0.38 | 26.0 [25.5-28.5] | 1.00 |
| Driving pressure (cmH2O) | PEEP_base_ | 9.5 [7.2-11.8] |  | 11.0 [8.7-11.8] |  |
|  | PEEP_set_ | 8.0 [6.5-10.2] | 0.54 | 11.8 [9.9-11.9] | 1.00 |
| Tidal volume (mL/kg PBW) | PEEP_base_ | 5.7 [5.4-6.7] |  | 7.0 [6.8-7.3] |  |
|  | PEEP_set_ | 5.6 [5.4-6.6] | 1.00 | 7.1 [6.8-7.4] | 0.95 |
| Static compliance (mL/cmH2O) | PEEP_base_ | 54 [48-62] |  | 38 [33-43] |  |
|  | PEEP_set_ | 64 [54-72] | 0.75 | 39 [36-42] | 1.00 |
| PaO2 (mmHg) | PEEP_base_ | 72 [59-86] |  | 90 [86-102] |  |
|  | PEEP_set_ | 66 [62-73] | 1.00 | 126 [99-129] | 1.00 |
| PaO2/FiO2 ratio (mmHg) | PEEP_base_ | 114 [64-171] |  | 225 [194-227] |  |
|  | PEEP_set_ | 112 [71-150] | 1.00 | 240 [222-260] | 1.00 |
| SpO2 (%) | PEEP_base_ | 94 [93-96] |  | 97 [96-97] |  |
|  | PEEP_set_ | 94 [94-95] | 1.00 | 96 [96-98] | 1.00 |
| PaCO2 (mmHg) | PEEP_base_ | 48 [46-53] |  | 44 [39-44] |  |
|  | PEEP_set_ | 53 [49-59] | 0.75 | 35 [32-40] | 1.00 |
| Systolic Blood Pressure (mmHg) | PEEP_base_ | 142 [113-165] |  | 117 [107-127] |  |
|  | PEEP_set_ | 151 [138-162] | 1.00 | 157 [152-162] | 1.00 |
| Diastolic Blood Pressure (mmHg) | PEEP_base_ | 63 [48-79] |  | 52 [51-52] |  |
|  | PEEP_set_ | 58 [46-75] | 1.00 | 66 [63-68] | 1.00 |
| Mean Arterial Pressure (mmHg) | PEEP_base_ | 83 [79-92] |  | 74 [70-77] |  |
|  | PEEP_set_ | 88 [79-101] | 1.00 | 96 [93-99] | 1.00 |
| Heart rate (/min) | PEEP_base_ | 74 [66-80] |  | 75 [68-96] |  |
|  | PEEP_set_ | 78 [66-88] | 1.00 | 71 [60-94] | 0.54 |

Data are presented as mean (standard deviation), count (%) or median [25th and 75th percentile]. * p < 0.05
